# Correlation between tibial valgus deformity and aspect ratio of resected tibial surface in female Chinese patients undergoing total knee arthroplasty

**DOI:** 10.3389/fsurg.2022.1079981

**Published:** 2023-01-06

**Authors:** Dejin Yang, Xiang-Dong Wu, Yixin Zhou, Xinghua Yin, Yong Huang, Hongyi Shao, Hao Tang

**Affiliations:** Department of Orthopaedic Surgery, Beijing Jishuitan Hospital, Fourth Clinical College of Peking University, Beijing, China

**Keywords:** alignment, morphology, aspect ratio, tibia, valgus knee, total knee arthroplasty

## Abstract

**Background:**

Morphology of the resected tibial surface is the reference for tibial component design, selection, and implantation in total knee arthroplasty (TKA). This comparative study sought to answer whether valgus deformity of the tibia would affect the morphology of the resected tibial surface in TKA.

**Methods:**

Thirty-one female Chinese patients with valgus tibias were retrospectively and consecutively identified from a single-center registration database. Thirty-one patients with well-aligned tibias were matched in terms of gender, height, and weight. Weight-bearing full-length radiographs and computed tomography images of the whole lower limb were obtained for every case. Tibial resection was mimicked perpendicular to the mechanical axis of the tibia in the frontal plane with 3° of posterior slope and a cut level individualized by the actual intraoperative cut. On the resected surface, mediolateral dimension (MLD), medial anteroposterior dimensions (mAPD), and lateral anteroposterior dimensions (lAPD) were measured, and aspect ratios (AR) were calculated. We compared the AR between the two groups.

**Results:**

The aspect ratio of resected tibial surface positively correlated with tibial valgus alignment. Patients with valgus tibias had significantly smaller AR (MLD/mAPD) for the medial plateau (1.50 ± 0.06 vs. 1.54 ± 0.07, *P* = 0.032). However, the AR for the lateral plateau was similar between the two groups (1.63 ± 0.08 vs. 1.65 ± 0.07, *P* = 0.328).

**Conclusion:**

This difference in morphology of resected tibial surface between valgus and well-aligned tibias should be considered in tibial component design, as well as in the selection and placement of TKA implants for knees with valgus tibias.

## Introduction

In total knee arthroplasty (TKA), the morphology of the resected tibial surface is the anatomical basis for component design and the reference for implantation. Tibial components should have proper shape and size to match the resected tibial surface. Also, the resected surface should be appropriately covered to provide sufficient bone support to the tibial component to prevent subsidence (1). Moreover, a proper tibial tray should be selected both in size and shape to avoid implant overhang, which further reduces iatrogenic irritation to the surrounding soft tissues (2). Bone-implant conformity in TKA is essential for proper rotational positioning of the implant and good clinical outcomes (1, 3–5).

In previous reports, bone-implant mismatch rates can be as high as 20% (1, 5–10). Potential underlying causes are the conflict between the limited number of implant components size, the wide variations in shape among ethnicities and individuals, and possible deformity-related differences. Ethnic and gender differences in the anthropometry of the proximal tibia have been frequently reported across a broad spectrum of different ethnicities. At the same time, the worldwide distributed manufacturers provide limited implant options both in size and shape, which may not cover the wide range of morphological differences between ethnicity and gender (9–16). Different tibial alignments/deformities may also contribute to the morphologic differences of the resected tibial surface. Some researchers suggested that the surface of the tibial plateau changes as osteoarthritic patients develop knee deformities (5). Furthermore, the level of tibial resection also influences the shape of the resected surface, which should be individualized for different alignments and joint deformities (17). Mori et al. measured the aspect ratio (AR, the ratio of the mediolateral (ML) dimension to the anteroposterior (AP) dimension) of the resected tibial surface in Japanese female patients with varus knees. They found that the aspect ratio of resected tibial surface was inversely correlated to the degree of tibia vara (18). However, to our knowledge, there is a paucity of literature available to ascertain whether the valgus alignment of the tibia affects the morphology of the resected tibial surface in TKA patients.

In this comparative study, we measured the AR of resected tibial surface on 3-dimensional (3D) computed tomography (CT) images at an individualized cutting level, which was coincident with the actual cut in TKA. In the present studies, we sought to answer whether the aspect ratio was different for the valgus tibia compared with the well-aligned tibia.

## Methods

This was a retrospective comparative study with matched groups. This study was approved by our institutional review board (IRB). All patients had signed the informed consent form with regard to the further use of their radiographic images in medical research without individual privacy exposure.

### Study population

The valgus tibia group was consecutively extracted from a single-center registration database. The inclusive criteria were: female patients with age above 45 years; TKA cases performed from January 2015 to December 2016; preoperative weight-bearing full-length radiographs of the lower limb (both AP view and lateral view), and CT volumetric data of the proximal tibia available in the database. Male patients were not included in this study, mainly due to the relatively low volume of male patients in the database. The exclusive criteria were: inflammatory arthritis which was frequently coincident with severe flexion contracture; post-traumatic arthritis or previous osteotomy on the tibia, which might influence the shape of the proximal tibia; the interval of the CT scan greater than 2 mm, which might lead to difficulties in discriminating cortical bone from osteophytes. From more than 3,000 TKA cases, 31 cases were finally qualified for the valgus tibia group. For comparison, 31 cases of the well-aligned tibia were matched according to the same inclusive and exclusive criteria, with height ± 5 cm, weight ± 10 cm, and age ± 10 years. Apart from the patient size, subjects were also matched by age because the relationship between age and tibial anthropometry was not clear yet. All CT scans were performed with an Aquilion sixty-four-slice spiral CT scanner (Toshiba, Otawara, Japan). All the demographic data was comparable between the two groups (Table 1).

**Table 1:** Aspect ratios of resected tibial surface with different tibial alignment.

	Valgus (*n* = 31)^†^	Well-aligned (*n* = 31)^†^	*P* value
Demographics			
Age/year	64.5 ± 6.3	62.6 ± 9.9	0.371
Height/cm	160.7 ± 4.2	159.8 ± 4.0	0.405
Weight/kg	66.2 ± 8.3	66.3 ± 7.0	0.947
MPTA/deg	93.0 ± 2.6	87.3 ± 1.0	<0.001
AR			
mAR	1.50 ± 0.06	1.54 ± 0.07	0.032
lAR	1.63 ± 0.08	1.65 ± 0.07	0.328

MPTA, medial proximal tibial angle; AR, aspect ratio; mAR, medial aspect ratio; lAR, lateral aspect ratio.

^†^ Values were given as mean ± SD.

### Prosthesis coverage simulation

**Figure 1:**
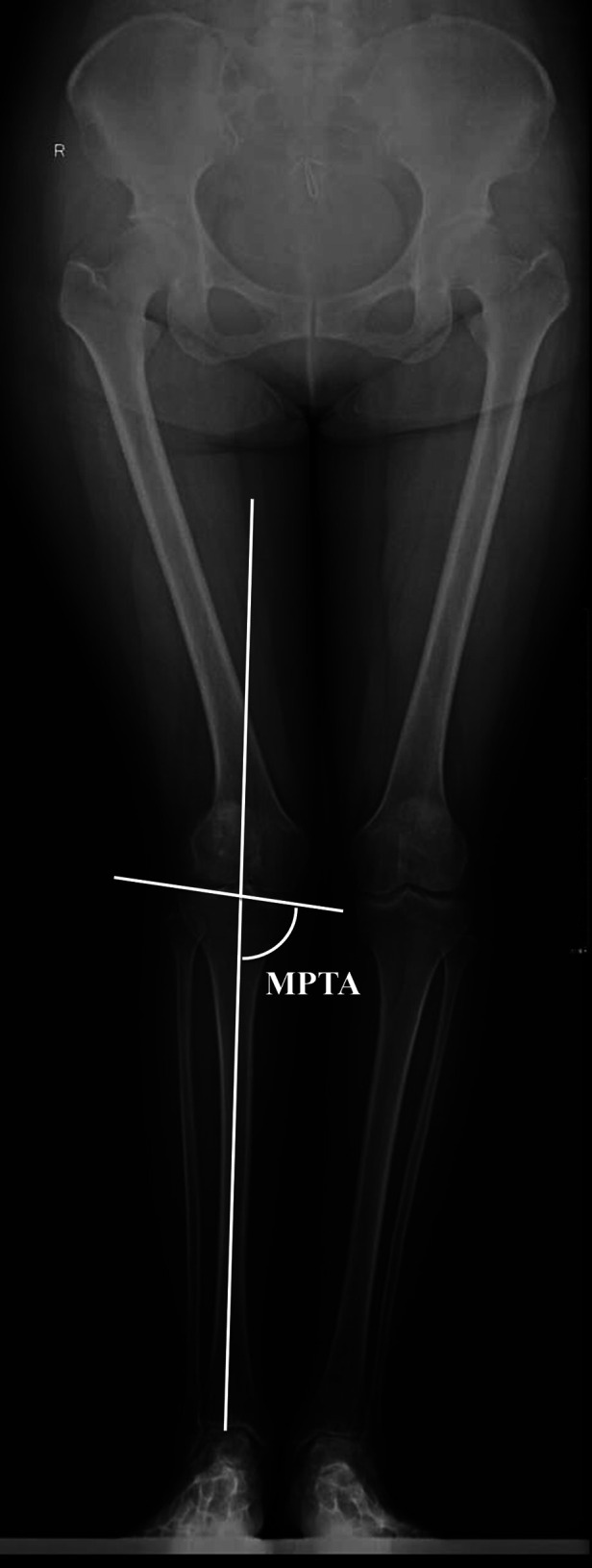
On anteroposterior view of the full-length x-rays, the medial proximal tibial angle (MPTA) was measured as the medial angle between a line connecting the medial plateau and lateral plateau and a line connecting the center of the tibial eminence and the center of the talar dome.

On AP view of full-length radiographs, the alignment of the proximal tibia was measured and recorded as the medial proximal tibial angle (MPTA) (Figure 1). MPTA was measured as the medial angle between a line connecting the medial and lateral plateau and the tibial mechanical axis (TMA), defined as a line connecting the center of the tibial eminence and the center of the talar dome on AP view x-ray. A valgus tibia was defined as a tibia with MPTA > 90°. A well-aligned tibia was defined as a tibia with 90°≥MPTA ≥ 85°.

The 3D computer program MIMICS (Version 16.0, Materialise, Belgium) was utilized to process the volumetric CT data and make radiographic measurements. Before measurements, the proximal tibial bone was repositioned with the mechanical axis as vertical in the frontal plane and the Akagi line in the sagittal plane. The Akagi line was previously defined as a line connecting the middle of the posterior cruciate ligament insertion site and the medial border of the patellar tendon insertion on the tibial tuberosity (19). Osteophytes were excluded from any measurements taken *via* segmentation processing in MIMICS.

**Figure 2:**
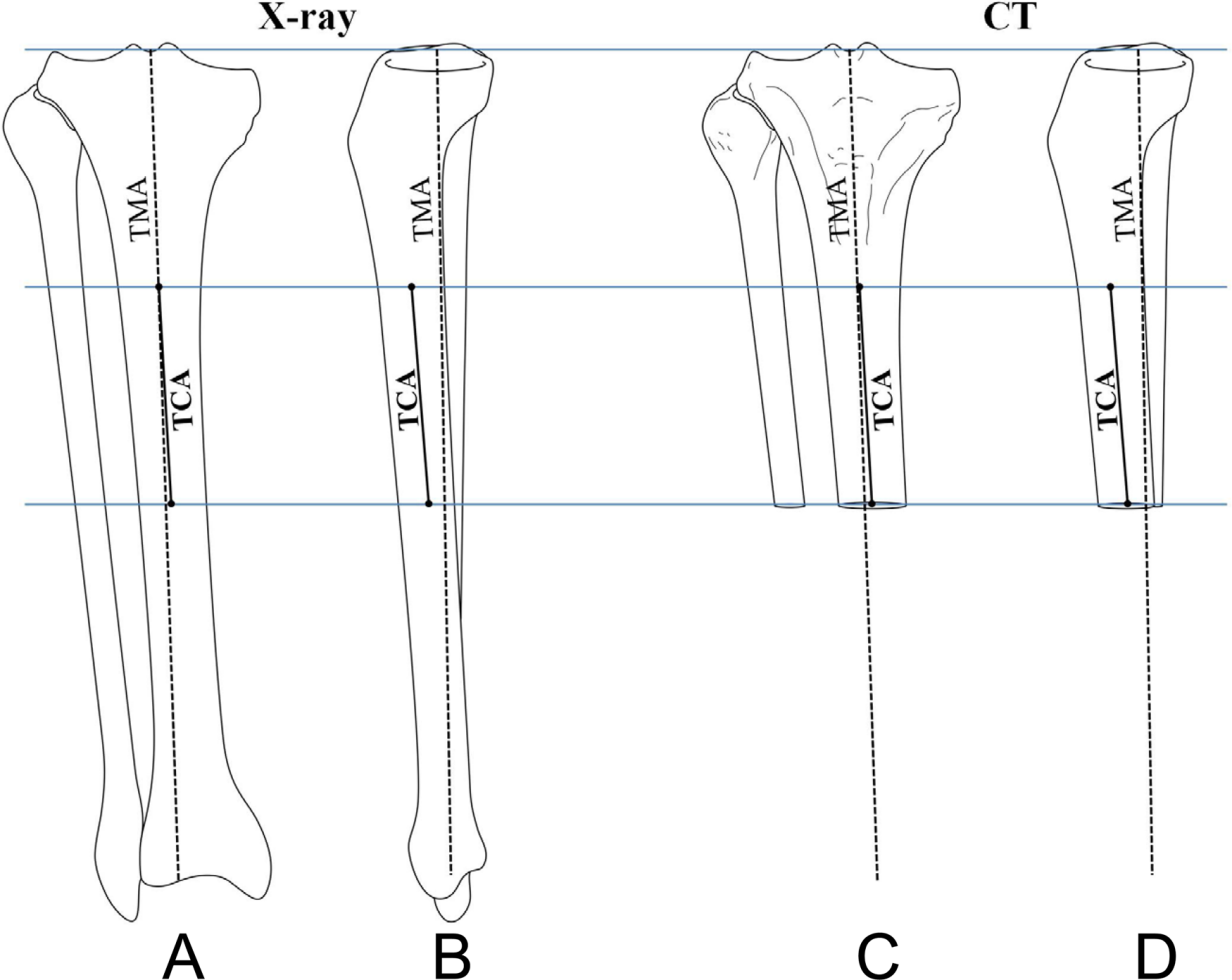
A uniform 4-step method was used to define the tibial mechanical axis (TMA) on 3D CT models. Step 1: TMA was drawn on both the AP and lateral views of the full-length radiographs (**A,B**). Step 2: The TCA of the proximal tibia was defined as a line connecting the centers of the canal at two distant levels. Then, the TCA was copied from the full-length radiographs to 3D CT models based on the same levels (**C,D**). Step 3: The intersection angles was measured between the TMA and the TCA both on the AP and lateral views of the full-length radiographs (**A,B**). Step 4: On 3D CT models, the TMA was defined based on the measured intersection angles and the copied TCA (**C,D**).

Given that the CT scan of the knee did not include the whole tibia bone, the tibial canal axis (TCA) of the proximal tibia was utilized as a reference to define TMA on 3D CT models of the tibia, for which a uniform 4-step method was followed (Figure 2).

Step 1: TMA was drawn on both the AP and lateral views of the full-length radiographs (18).

Step 2: The TCA of the proximal tibia was defined as a line connecting the centers of the canal at two distant levels. Then, the TCA was copied from the full-length radiographs to 3D CT models based on the same levels.

Step 3: We measured the intersection angles between the TMA and the TCA on both the AP and lateral views of the full-length radiographs.

Step 4: On 3D CT models, the TMA was defined based on the measured intersection angles and the copied TCA.

The tibial resection was mimicked as a cut vertical to the TMA in the frontal plane with a 3° posterior slope in the sagittal plane. The mimicked cut level was individualized by the actual intraoperative cut level, which was extracted from the medical record. We individualized the tibial resection level to control the reported effect of the cut level on the shape of the resected surface. No reduction osteotomy was mimicked on the edge of the tibial plateau as we could not imitate soft-tissue balancing like actual surgeries.

**Figure 3:**
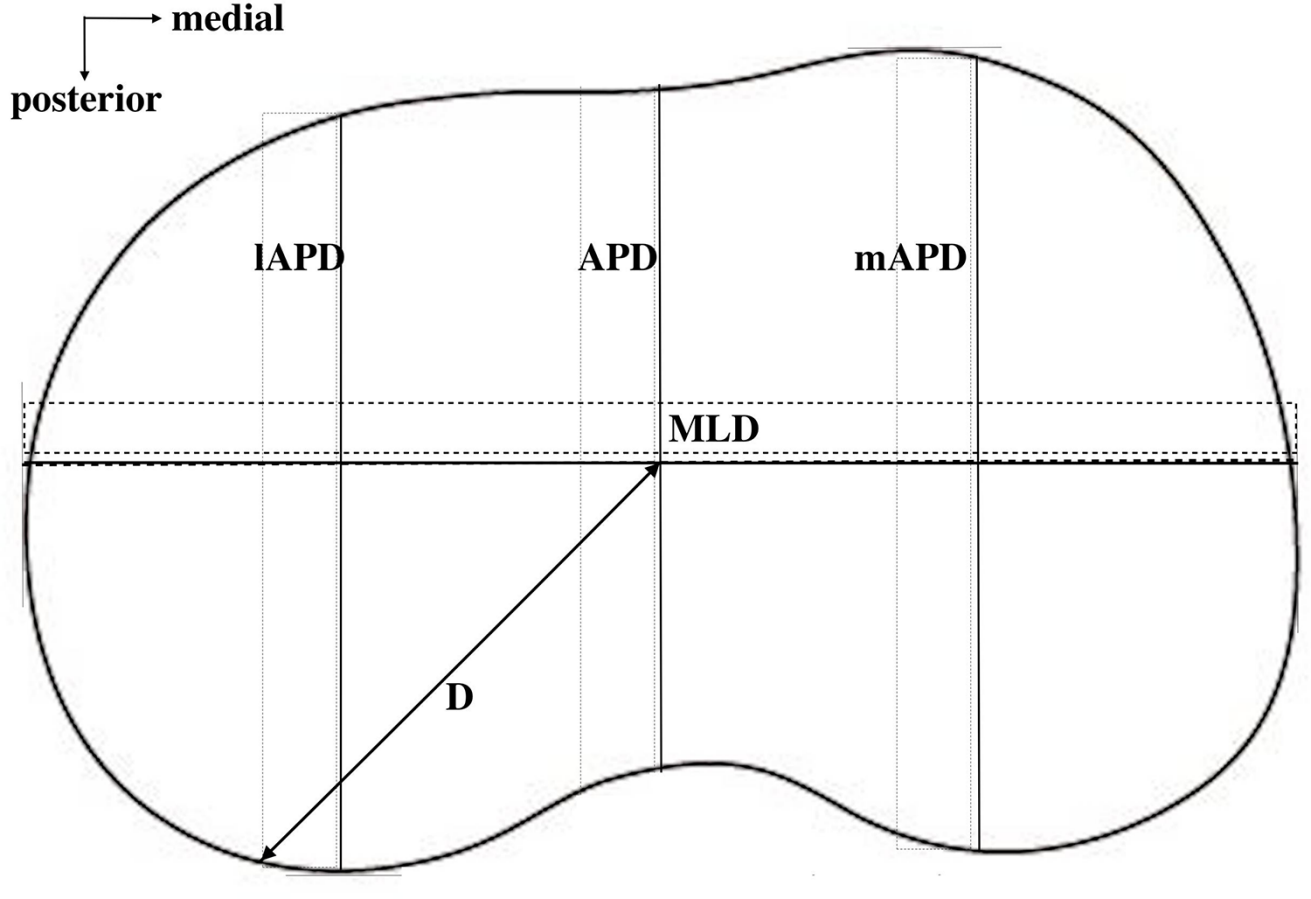
Different dimensions on the resected tibial surface were measured. MLD, mediolateral dimension; mAPD, medial anteroposterior dimension at the middle point of the medial half of the whole surface; lAPD, lateral anteroposterior dimension at the middle point of the lateral half of the whole surface.

On the resected surface, the ML dimension (MLD) of the tibial bone was measured (Figure 3). Then, the medial anteroposterior dimension (mAPD) was measured through the medial 25% point on MLD, and the lateral anteroposterior dimension (lAPD) was measured through the lateral 25% point on MLD (Figure 3). These measurements were then put into the calculation of AR: the medial AR (mAR = MLD/mAPD) and the lateral AR (lAR = MLD/lAPD).

### Statistical analysis

The Kolmogorov-Smirnov test was utilized to confirm whether the demographic data and measurement data were normally distributed. The independent *t*-test was used to compare the two groups. A *P*-value less than 0.05 is considered statistically significant. Statistical software SPSS (Version 24.0, SPSS Inc., Chicago, IL, USA) was used for analysis.

## Results

There were no statistically significant differences for any of the demographic data (age, height, and body weight) between the two groups (*P* > 0.10).

Tibial valgus alignment influenced the morphology of the resected tibial surface (Table 1). Valgus tibias had significantly smaller mAR than well-aligned tibias (1.50 ± 0.06 vs. 1.54 ± 0.07, *P* = 0.032). A similar trend of difference was shown between the two groups for the lAR, but the difference was not statistically significant (1.63 ± 0.08 vs. 1.65 ± 0.07, *P* = 0.328).

## Discussion

Bone-implant shape conformity of the tibial component to the resected tibial surface in TKA is important for attaining proper rotational positioning of the implant and good clinical outcomes (3, 4). In this regard, morphometric data of the resected tibial surface contributes to guiding tibial prosthesis design and selection. Many other factors, including ethnicity and gender, also have an effect on the morphology of the resected tibial surface (9–16). However, the effect of tibial valgus deformity on the morphology of the resected proximal tibia remains unclear.

The major finding of the current study was that the valgus deformity of the proximal tibia significantly influenced the morphology of the resected tibial surface. More specifically, valgus tibias had smaller mAR than well-aligned tibias (1.50 vs. 1.54). The data also indicated that valgus tibias had smaller lAR than well-aligned tibias (1.63 vs. 1.65), despite no statistically significant difference. Both smaller mAR and lAR suggested relatively narrow tibial surfaces for knees with tibial valgus deformity. This difference suggests that awareness of both implant selection and placement for the valgus tibias is indicated. In theory, a prosthesis with a narrow tibial baseplate should be selected, which allows greater implanting freedom and better bone coverage. Given that the variation in valgus knee types results from different deformity origins, attention should be given to the tibia when preparing and placing the tibial component for valgus knees. The shape of the resected surface could be much narrower by lateral reduction osteotomy, which is potentially needed for valgus knees. This correlation and trend of difference were similar to the previous results for tibia vara (18). In order to obtain a vertical cut according to the mechanical alignment method, both valgus and varus deformity of the tibia would drive the surgeon to make a more oblique cut from the tibial plateau. The steep metaphyseal outer contour of the proximal tibia probably contributes to these differences between different oblique cuts, or even between different cut levels, as reported (17).

A narrower tibial cutting surface in the valgus tibia could mislead surgeons either to choose a smaller-sized tibial component than expected or to leave the component overhang medially or laterally. It also predisposes surgeons toward mal-rotating the tibial component to avoid overhang. Clary et al. also reported that certain tibial anthropometrics might predispose particular patients to internal malrotation of the tibial base (3).

A broad spectrum of tibial aspect ratios from different alignment and cutting levels makes an appeal for more accommodating shapes and sizes for any set of devices. Whether symmetrical, asymmetrical, or anatomical designs would provide better coverage remains controversial. Dai et al. reported that an anatomic design demonstrated a meaningful increase in tibial coverage with accurate rotational alignment compared to both symmetric and asymmetric designs (20). Incavo et al. compared eight different tibial implant designs with 35 resected tibias, and found that the six symmetrical designs provided more coverage than the two asymmetric designs (7). Westrich et al. compared one asymmetrical tibial tray design with two different symmetrical tibial tray designs, and found that the shape of the tibia at the resection level was asymmetrical. However, the authors acknowledged that the actual shape of the tibial tray and the number of accommodating sizes provide the best ability of a TKA implant system to maximize tibial coverage, and not simply an asymmetrical or symmetrical design (6). Based on our study findings, additional implant options with narrow tibial baseplates may better fit patients’ anatomical variations, maximizing the resected surface coverage. A recent multicenter study on the resected femoral surface concluded that an additional mediolateral implant option improves component fit across ethnicities (21).

### Limitations

This study had several limitations. First, the measurements taken in the current study were imaging-based and not performed intraoperatively; thus, measured resection may not correspond to exactly the same shape as the actual bone cut. Reduction osteotomies may be performed intraoperatively for severe valgus tibias, which undoubtedly will change the aspect ratio of the resected surface. However, a reduction osteotomy would further lead to a narrower tibial surface and thus reduce the aspect ratio, which would not change our conclusions. Second, we did not include male patients or patients of other ethnicities, besides female Chinese patients. Given that differences have been reported previously among variations in gender and ethnicity, the conclusion of the current study should be generalized to other patient populations with caution.

## Conclusion

In conclusion, tibia valgus deformity affects the morphology of the resected tibial surface in TKA. Valgus tibias have a narrower resected tibial surface than well-aligned tibias. This difference should be taken into consideration in tibial component design, as well as implant selection and placement in TKA for knees with tibial valgus deformity.

## Data Availability

Publicly available datasets were analyzed in this study. This data can be found here: The datasets used and/or analyzed during the current study are available from the corresponding author on reasonable request.
